# Józef Polikarp Brudziński (1874–1917)

**DOI:** 10.1007/s00415-018-8854-3

**Published:** 2018-04-04

**Authors:** Krystyna Makowska, Kamila Szymanska

**Affiliations:** Department of Clinical Physiology, Faculty of Veterinary Medicine University of Warmia and Mazury in Olsztyn, ul Oczapowskiego 13, 10-718 Olsztyn, Poland

**Fig. 1:**
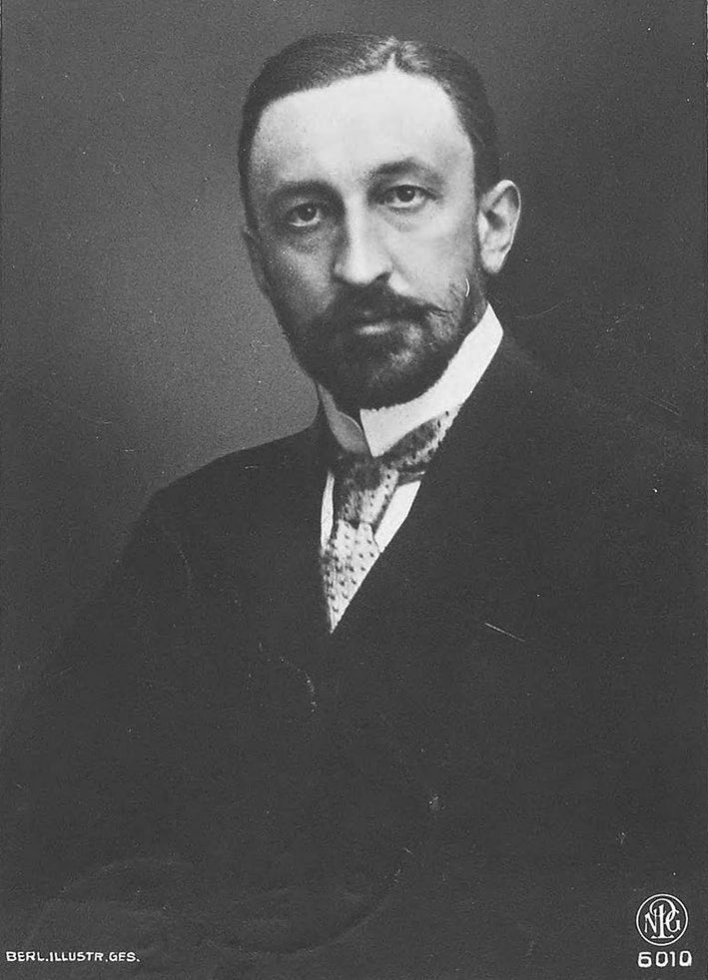
Józef Polikarp Brudziński. Photo from public domain

Józef Polikarp Brudziński (Fig. 1) was born into a noble family with patriotic traditions on 26 January 1874 in Bolewo in Płock Governorate [1]. After finishing middle school in Warsaw, Brudziński started his medical studies at the University in Dorpat (currently Tartu in Estonia) in 1891, but in 1894 he moved to Moscow, where he received his medical degree in 1897 and decided to specialize in pediatrics [2]. After graduation, Brudziński went for a 3-year foreign internship. At first, he worked in a pediatric clinic in Cracow (situated at that time in Austro-Hungarian Empire), and then he continued his education in Graz, Paris, London, Berlin and Vienna. During the internship, Brudziński more than once worked with world-renowned scientists and surgeons, including Theodor Escherich, Jacques Granscher and Antion Marfan [3].

Brudziński returned to Warsaw in 1900 and took a job in Rev. G. P. Baudouin’s Foster Care Centre at Warsaw’s Infant Jesus Hospital [3, 4]. In 1903, he received a proposal to take up the position of a general surgeon at the newly built Anna Maria Pediatric Hospital in Lodz. Brudziński again went abroad for a couple of months to learn about organization and operating procedures of modern pediatric hospitals in Europe. He started work at Anna Maria Hospital in 1905 [3]. Not long afterward, this hospital became an important scientific center in Polish lands.

Brudziński worked at Anna Maria Hospital until 1910, when he received an invitation to participate in the design and building of a new modern pediatric hospital in Warsaw from the foundation of Zofia Szlenkierówna, the pioneer of nursing in Poland. Before the move to Warsaw, Brudziński again went away on a tour of European pediatric hospitals for a few weeks. Building of the pediatric hospital in Warsaw, named after the parents of the founder, Karol and Maria Szlenkier Hospital, started in 1911, and in 1913 the first patients were admitted.

Brudziński directed the hospital until 1915, when he became involved in the reactivation of Polish higher education in Warsaw to replace the Russian language Imperial Warsaw University, which was moved to Rostov-on-Don at the beginning of the First World War [1]. Brudziński was elected rector of Warsaw University on 2 November 1915, and in 1916 he started lectures with students in the newly created medical faculty [2]. Unfortunately, the hard work impaired Brudziński’s health. He died of nephritis and uremia in Warsaw on 18 December 1917 at the age of only 43 years.

Józef Brudziński was not only a pediatrician, but also a scientist. He was the author of 55 scientific articles published in Polish and European medical journals, including “Gazeta Lekarska”, “Medycyna”, “Wiener klinische Rundschau”, “Revue Neurologique” and “Terapie der gegenwart” [3, 4]. Articles by Brudziński dealt with nutrition, bacteriology of the gastrointestinal tract, various contagious childhood diseases and organization of work in the hospital [1, 4]. However, the most important scientific works by Brudziński relate to neurology [1, 4].

During his medical practice at Anna Maria Hospital in Lodz, Brudziński observed and described symptoms specific to meningitis. In 1908, he published in the pages of “Medical Review” (original Polish title “Przegląd Lekarski”) his first work concerning this subject [5], where he described “contralateral reflex symptom” occurring mainly in hemiparesis but also during meningitis. This sign, according to Brudziński, consisted of the straightening of a paralyzed limb after passive flexion of the contralateral healthy limb. His next article, published in 1909 in “Pediatric Review” (original Polish title “Przegląd Pediatryczny”) [6], described a neck sign in meningitis, in which forced flexion of the neck causes a reflex flexion of the hips. His final publication about signs in meningitis was published in 1916 in “Medical Gazette” (original Polish title “Gazeta Lekarska”), describing cheek and symphyseal signs [1, 3]. In the first of these, pressure on the cheek elicits a reflex rise and flexion of the forearm, and in the second pressure on the pubic symphysis elicits reflex flexion of the hip and knee and abduction of the leg. Currently, these are known as “Brudziński’s signs” and are described in many neurology textbooks, particularly the neck sign.

It should be pointed out that Brudziński not only described the above-mentioned signs, but also worked on the explanation of their mechanisms. For this purpose, he performed numerous experiments on laboratory animals at the Institute of Physiology at the Jagiellonian University in Cracow, under the direction of the physiologist Napoleon Cybulski [1]. Brudziński’s commitment to scientific work and discoveries were highly valued in his own time. He was often invited to provide lectures during the meetings of various medical societies and received numerous awards. Moreover, Brudziński set up the scientific journal “Pediatric Review”, the first specialized journal concerning the childhood diseases in Central and Eastern Europe. In recognition of Brudziński’s achievement in pediatric neurology, in 1909 the Jagiellonian University in Cracow awarded him the degree of doctor of medicine without the required examinations [1]. Apart from clinical, scientific and organizational talents, Brudziński showed interests in teaching, community service, and political and patriotic activity [4].

The attitude of Brudziński toward work is reflected most clearly in the words of Rector Kostanecki from Cracow, who said at the funeral of Brudziński: “He went to his creative work as a knight into battle… he went to work with the singing in the soul… he went with Polish fantasy” [1]. In turn, students of Warsaw University wrote in Brudziński’s obituary: “He built in days of turmoil and despair. He embraced with his ardent heart the Past, Present and Future of the Nation” [3, 4].
